# AMRViz enables seamless genomics analysis and visualization of antimicrobial resistance

**DOI:** 10.1186/s12859-024-05792-9

**Published:** 2024-05-16

**Authors:** Duc Quang Le, Son Hoang Nguyen, Tam Thi Nguyen, Canh Hao Nguyen, Tho Huu Ho, Nam S. Vo, Trang Nguyen, Hoang Anh Nguyen, Minh Duc Cao

**Affiliations:** 1 AMROMICS JSC, Nghe An, Vietnam; 2 Faculty of IT, Hanoi University of Civil Engineering, Hanoi, Vietnam; 3 Oxford University Clinical Research Unit, Hanoi, Vietnam; 4 Bioinformatics Center, Institute for Chemical Research, Kyoto University, Kyoto, Japan; 5 Department of Medical Microbiology, The 103 Military Hospital, Vietnam Military Medical University, Hanoi, Vietnam; 6 Department of Genomics and Cytogenetics, Institute of Biomedicine and Pharmacy, Vietnam Military Medical University, Hanoi, Vietnam; 7 Center for Biomedical Informatics, Vingroup Big Data Institute, Hanoi, Vietnam

**Keywords:** Microbial genomics, Visualization, Antimicrobial resistance

## Abstract

We have developed AMRViz, a toolkit for analyzing, visualizing, and managing bacterial genomics samples. The toolkit is bundled with the current best practice analysis pipeline allowing researchers to perform comprehensive analysis of a collection of samples directly from raw sequencing data with a single command line. The analysis results in a report showing the genome structure, genome annotations, antibiotic resistance and virulence profile for each sample. The pan-genome of all samples of the collection is analyzed to identify core- and accessory-genes. Phylogenies of the whole genome as well as all gene clusters are also generated. The toolkit provides a web-based visualization dashboard allowing researchers to interactively examine various aspects of the analysis results. Availability: AMRViz is implemented in Python and NodeJS, and is publicly available under open source MIT license at https://github.com/amromics/amrviz.

**Supplementary Information:**

The online version contains supplementary material available at 10.1186/s12859-024-05792-9.

## Introduction

With the advances of high-throughput sequencing, the number of bacterial genomes sequenced has increased exponentially over the last decade. Studying the genomics of pathogenic bacteria, especially those with antibiotic resistance can provide insights into the development and transmission of antibiotic resistance. Pan-genome analysis [1] has increasingly become the method of choice for population genomics. Here, the pan-genome refers to the entire set of gene families of a given clade; core genes, the set of gene families presenting in the majority of isolates in the clade, are considered to characterize the clade whereas accessory genes, those present in a small number of organisms, provide a view of the variation and evolutionary trajectories [2]. Pan-genome analysis is particularly relevant to microbial genomics because of the plasticity of their genomes; they can rapidly acquire genes from other organisms, especially in an environment with evolutionary pressure such as with antibiotics [3]. The ability to study the complete genetic information of a collection of large number of bacterial genomes also provides the potential to generate insights into the pathogenic genotype/phenotype relationships [4, 5], pathogenic virulence transmissibility [6] and antibiotic resistance tracking [7].

While there are numerous software tools for analyzing bacterial genomics data, for example [8–11], they are often designed for a specific task of the large analysis pipeline. These tools often require bioinformatics expertise to set up, and to connect them together to make up an end-to-end pipeline. Many tools require users to resort to multiple external websites such as [12–14] for the analysis. As a result, bacterial genomics analysis, especially for a collection of hundreds of samples, involves many manual steps that can be time-consuming and error-prone. Recently, several computational pipelines have been developed by stitching the analysis tools into a workflow to facilitate the analysis of microbial genomic data, notably Nullarbor [15], Bactopia [16], and ASA^3^P [17]. However, these software pipelines usually require high-end computational infrastructures and are not scalable for large collections of samples [18]. In addition, there is a lack of integrated tools for visualization and interpretation of analysis results.

To address these shortcomings, we developed AMRViz, a software package to manage the analysis of drug-resistant bacteria collections. AMRViz is bundled with an end-to-end analysis pipeline consisting of the considered best practice methodologies for microbial genomics to streamline data processing. AMRViz also provides a comprehensive visualization of important aspects of the analysis and a dashboard to manage samples and collections. The analysis of a collection of bacterial samples can be initiated with a single command line. The analyses and visualization will be generated automatically. AMRViz is cross-platform, and can be conveniently installed, managed and operated with modest hardware requirements and minimum bioinformatics support.

## Methods and implementation

AMRViz toolkit consists of three components, namely (1) a pipeline for genomics analysis of a bacterial collection, (2) a platform for visualization of the analysis results, and (3) a system to manage samples and collections.

### Analysis pipeline

**Fig. 1:**
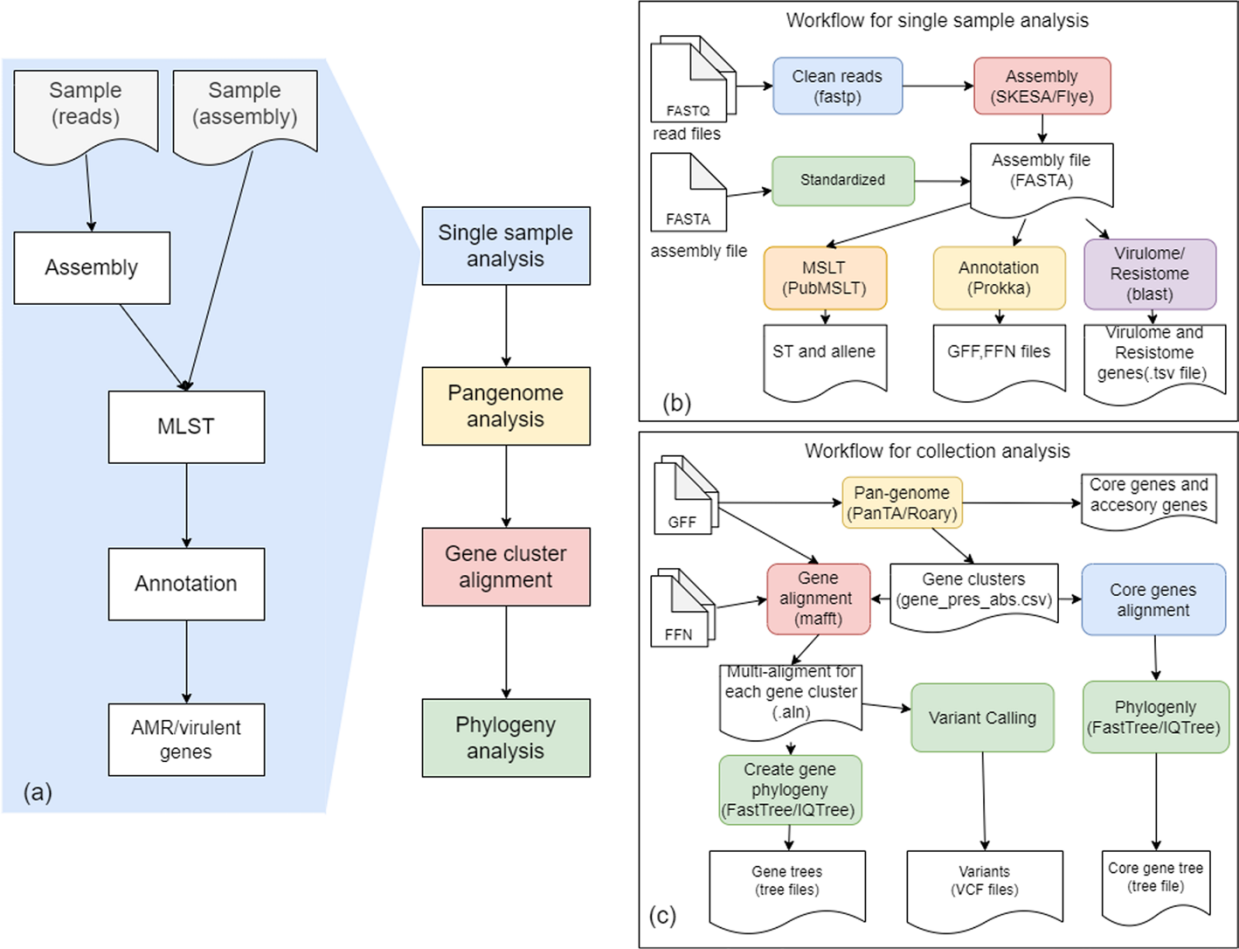
Schematic description of the AMRViz analysis pipeline. **a** Overall workflow; **b** workflow for single sample analysis, and **c** workflow for collection analysis

The analysis pipeline takes in as input a list of samples for a collection, and performs genomics analysis on each single sample as well as pan-genome analysis on the collection. The samples are listed in a tsv file, one sample per line. Metadata associated with the samples such as phenotypic information can be included in the tsv file. Data input for each sample can be in the form of sequencing data (fastq format), assembly sequence (fasta format) or annotation (gff3 format). AMRViz supports the most common sequencing technologies including Illumina, Pacbio and Oxford Nanopore.

Under the hood, AMRViz utilizes AMRomics [18], an efficient workflow to generate analysis results. Briefly, AMRomics performs single sample analysis for each sample, and then performs collection analysis for the collection (Fig. 1a). If the input data for a sample are sequencing data, the pipeline applies the appropriate assembly algorithm for the sequencing technology: SKESA [8] or SPAdes [9] for Illumina data and Flye [19] for Pacbio and Oxford Nanopore data (Fig. 1b). The assembly of the sample is then annotated with Prokka [10] to identify protein coding genes and transfer RNA sequences and their functions. The sample assembly is also subject to antibiotic resistance and virulence gene analysis using BLAST [20] against major relevant gene databases, and to multi-locus strain typing analysis with pubMLST database [12].

After all samples in the collection have undergone single sample analysis, the pipeline performs genomics analysis of the collection (Fig. 1c). Specifically, PanTA [21] or Roary [11] is used for pan-genome analysis of the collection to identify core- and accessory- genes. For each gene cluster, the gene sequences are translated to amino acid sequences which are used to generate the gene multiple sequence alignment (MSA) using MAFFT [22]. The alignment is performed on protein sequences to make use of the functional information encoded in protein sequences. Subsequently, the nucleotide MSA of the gene is inferred from the protein alignment and is used for phylogenetic analysis with FastTree 2 [23] or IQ-TREE2 [24] to utilize the information in the synonymous mutations. In addition, AMRViz builds the phylogeny of the collection from the concatenation of the MSAs of all core genes. Finally, AMRViz calls variants of all the samples in the collection against the reference pangenome, termed pan-SNPs, a novel concept introduced in AMRomics [18]. The variant profile of a sample is reported in a VCF file.

### Visualization platform

**Fig. 2:**
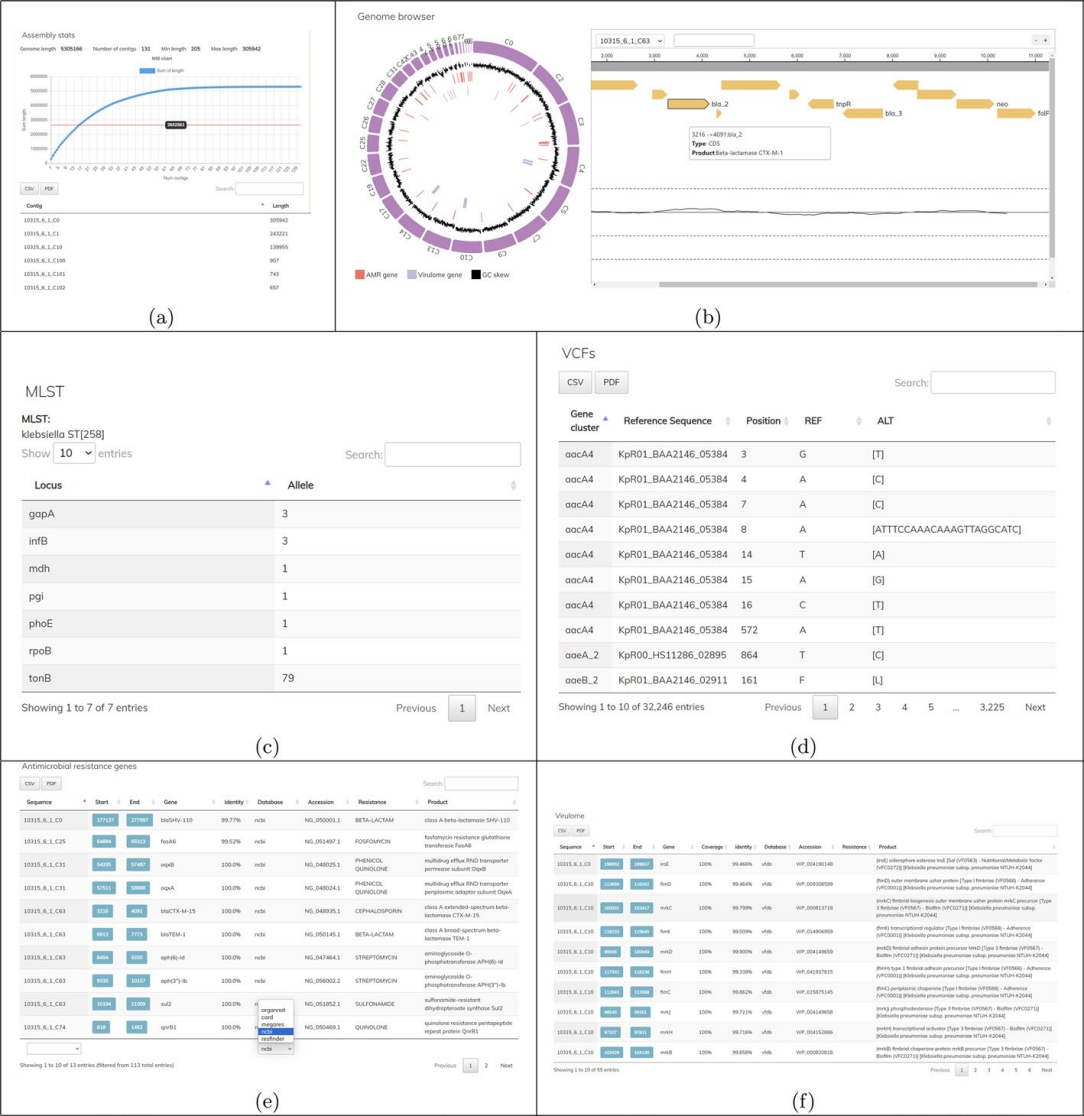
Visualization of the single analysis of a sample. **a** Showing of the assembly statistics. **b** Depiction of the genome as a circos and the genome browser. **c** MLST profile of the sample. **d** Listing of antimicrobial resistance genes, users can select the source of resistance gene database to show. **e** Listing of virulence genes

**Fig. 3:**
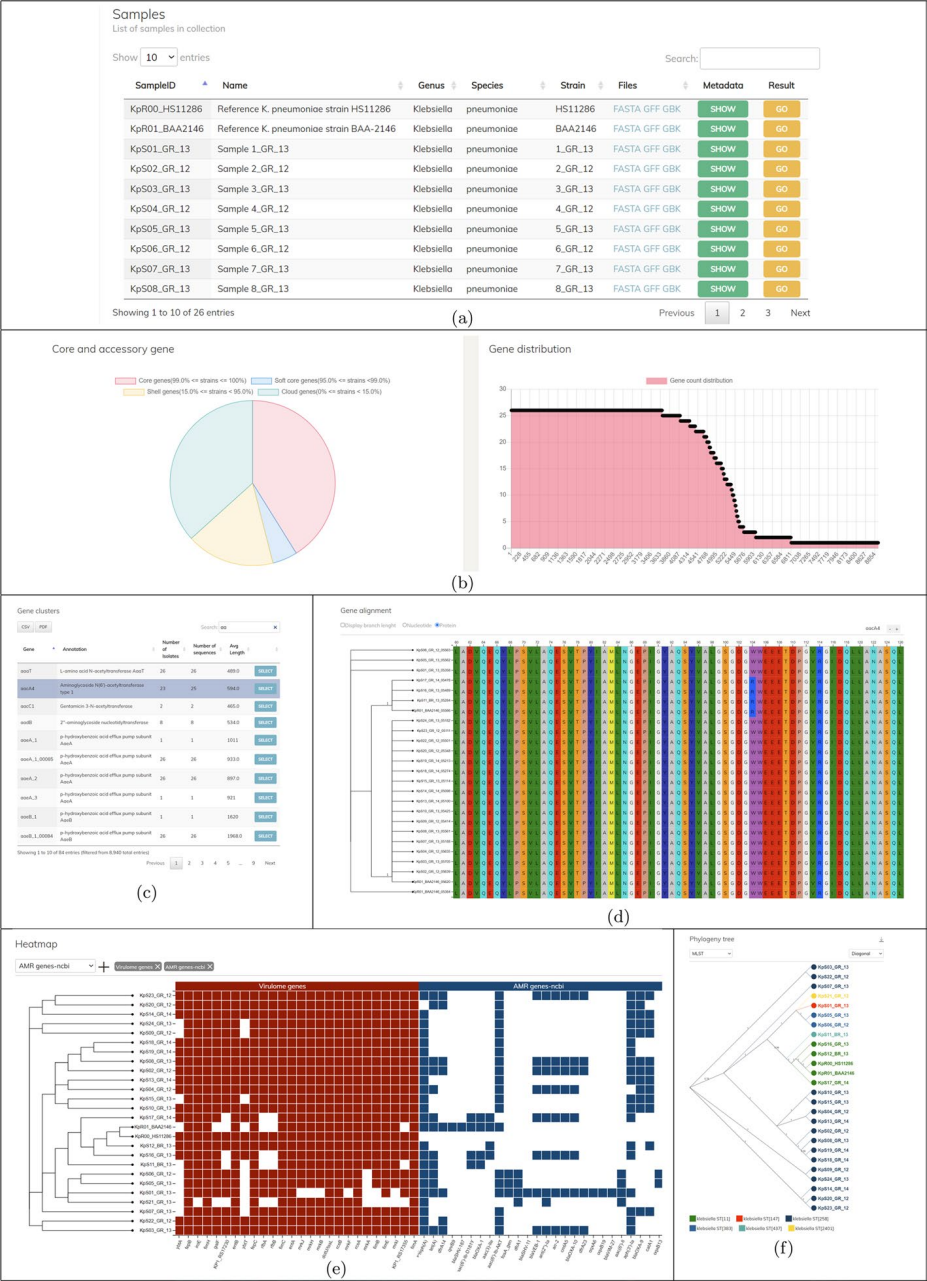
Visualization of the analyses for a collection. **a** Visualization of the statistics of core- and accessory genes and the gene distribution. **b** Listing of the gene families in the collection. **c** The multiple alignment of a gene family is displayed when a gene family is selected. **d** The heatmap showing the presence and absence of resistance and virulence genes. **e** The phylogeny of the samples in the collection

The platform exports all analysis results to a web server for visualization via a web browser. For each sample, AMRViz displays and visualizes detailed genomic information allowing users to interactively investigate the genomics analysis results (Fig. 2). Specially, the assembly statistics and a chart showing the length distribution of the contigs are presented (Fig. 2a). AMRViz also presents the genome structure of the sample via a circos chart and a genome browser, allowing users to have both an overview and detailed views of the genome (Fig. 2b). From the circos chart, users can view the genome structure, the contigs and their CG content, and the high-level locations of resistance genes and virulence genes on the genome. Users can select a contig or a gene of interest presented in the genome browser. AMRViz also shows the sample’s metadata, gene annotation, MLST profile (Fig. 2c) and variant against the pan-genome (Fig. 2d). Resistance genes and virulence genes are displayed in tables with paging and sorting for easy navigation (Fig. 2e, f). As AMRViz uses several databases of resistance genes for resistome analysis, users can select which source of resistance genes to display. Users can also search for resistance genes and virulence genes with keywords via the search boxes. When users select one of the resistance/virulence genes, the genome browser is zoomed to the location of the selected gene and its contig, enabling visual inspection of the gene and the vicinity of its position.

The pan-genome analysis of the whole collection is also be visualized, allowing interactive detailed investigation (Fig. 3). At the top of the page, AMRViz lists all samples in the collection (Fig. 3a). In this section, users can sort and search the list to find the sample of interest, view its metadata as well as access its single sample analysis result. AMRViz summarizes statistics of the core- and pan-genes in a pie chart and a bar graph (Fig. 3b). It also lists all gene families in a table (Fig. 3) with sorting and searching functions. When a gene family is selected, the multiple alignment of the gene is displayed (Fig. 3d). Users can toggle between the nucleotide and protein modes for the multiple alignment and zoom into the columns of interest for a closer inspection. A gene phylogeny is presented on the side of the alignment. The phylogeny of the gene can also be visually compared to the core-gene phylogeny. User can observe the difference between the two trees if there is a crossover between the inheritance links in the gene tree (data not shown).

AMRViz also produces a heatmap showing the presence of resistance genes and virulence genes together with the phenotype and metadata provided in the input, allowing visual inspection of any associations between gene presence and phenotypes (Fig. 3e). The phylogeny tree from the core-gene alignment of all samples in the collection is also generated (Fig. 3f). This tool displays a full range of tree information in various styles and bootstrap values. Furthermore, users can highlight samples with different colors based on metadata. When a node is selected on the tree, its child-node samples will be highlighted on the heatmap and alignment tool.

The web application is developed on the VUE framework (https://vuejs.org) with well-modularized visualization tools and is easy to integrate with other systems. The visualization tools are built on top of the following graphics libraries and tools: chart.js (https://www.chartjs.org/), d3js (https://d3js.org), phylocanvas (http://phylocanvas.org), circosJS (https://github.com/nicgirault/circosJS).

### Sample and collection management system

AMRViz provides a web-interface dashboard to manage dataset collections. Users can view and navigate all collections that have been analyzed. From the dashboard menu, users can also view the list of samples in the system. Both the collection and sample list views allow sorting by various fields and searching by keywords. They also provides access to analysis results and visualization for each collection/sample. Collections and samples can be added and updated with pipeline runs as described in the Analysis pipeline section.

## Case study

### A collection of clinical *Klebsiella pneumoniae*

We demonstrate AMRViz’s ability to perform an end-to-end automatic analysis and to visualize analysis results of a genome collection with two case studies. In the first case study, we used the tool to re-analyze a dataset including 89 samples of *Klebsiella pneumoniae* collected from major hospitals in Kathmandu [25]. We included in the collection two additional reference genomes with Genbank accessions GCF_000240185 and GCF_000364385 in fasta format. While the pipeline did not require a reference genome as part of the analysis, including one or several reference genomes enables the use of known genes in the construction of the pan-reference genome [18]. We also included 11 samples that were sequenced by Oxford Nanopore and a sample by Pacbio technologies to show AMRViz’s capability to handle the heterogeneity of data. Altogether, the collection contained 103 samples in various input types including sequencing assembly, short read and long read sequencing data. The details of the data of the case study are provided in *Availability of data and materials*.

Both the web server and the analysis were run on a laptop computer with a 20 hyper-thread CPU (Intel Core i7-1280P) and 32Gb of memory, running Ubuntu Linux 22.0. With one command line, we were able to process the whole dataset through the pipeline. The analysis took in total 12.54 h and consumed 10.01 Gb of memory to perform all analysis steps and import results into the web portal.

AMRViz was able to replicate the analysis performed by [25] as described in the original paper. We note that the MLST types were identical to the original paper, except that the cluster of ST1559 was identified as ST3395. We manually checked the sequences of the housekeeping genes for ST3395 in the MLST database and found they are identical to the genes in the cluster, thus confirming the accuracy of AMRViz. To ascertain that the discrepancy is not due to the error during assembly, we downloaded the assembly of one the samples in the cluster from Refseq database (Refseq accession GCF_000943155.1) and ran MLST typing using both our system and MLST server [26]. Again, both systems return ST3395 with 100% identity.

From the dashboard of the analysis results, AMRViz showed on the phylogeny diagram the formation of two major genetically distinct lineages, ST15 and ST3395 (Additional file 1: Figure S7). According to the results from [25] the ST15 isolates have S83F and D87A mutations in the gyrA gene and the S80I mutation in the parC gene. Using the alignment view tool (Additional file 1: Figures S3 and S4), these mutations can be easily seen when comparing the amino acid sequences of the isolates with the reference MGH78578. Furthermore, the gene tree comparing tool (Additional file 1: Figure S5) allowed us to see that among the ST15 isolates, 10315_6#28 has the gyrA gene closest to the reference. As shown in alignment view tool, 10315_6#28 has one less SNP than the rest of the ST15 isolates. For finding resistance genes, we used the AMRViz scanned across resistance gene databases such as ncbi, card, megares, argannot and found most of the resistance genes mentioned in [25]. The heatmap showed the relationship between resistance genes and drug resistance of isolates, by displaying both the presence of resistance genes and the resistance phenotype of each isolate. As an example, the relationship between *carbapenem* resistance and the bla-NDM1 gene was displayed (Additional file 1: Figure S6). Most of the isolates in the ST15 group with the bla-NDM1 gene were resistant to Imipenem and Meropenem.

For detailed analysis of the results of a single isolate, AMRViz provided a set of tools to visualize the result data in the form of interactive tables and graphs. As an example, the statistics of the assembly for sample 10315_6#17 was displayed (Additional file 1: Figure S1). The statistics included the number of contigs, the length of each contig and N50 statistics. The results of MLST analysis showed that this sample has ST 15. The list of resistance genes and virulence genes were presented in two interactive tables showing the sample harbored multiple resistance genes such as blaSHV, bla-NDM1,blaCTX-M-15, qnRB1, mrSE,armA, sul1,aadA2, strA, strB. We also found additional resistance genes that have not been reported previously in [25] such as fosA and tet(D). We could see the elements around this gene when looking for the location of gene bla-NDM-1 in this isolate using the genome browser (Additional file 1: Figure S1B).

### A set of *Streptococcus pyogenes* from Victoria, Australia

The gram-positive cocci *S. pyogenes*, or group A streptococcus (GAS), linked with seasonal scarlet fever, has increasingly caused invasive infection (iGAS) in many Australian states over the past decades. There are ongoing efforts in genomic surveillance from national public health labs to understand the epidemiology and upregulated virulence factors of iGAS samples around the country [27, 28]. For demonstration purposes, we extract a small set of 72 samples from the project PRJNA872282—sequencing of *Streptococcus pyogenes* *emm1* from Victoria and Queensland between 2005 and 2020. Together with 4 other *S. pyogenes* assemblies, we ran the pipeline on this dataset and the results were added to the collection dashboard of the visualization interface for browsing. The analysis took 2.16 h on the aforementioned computer, requiring 9.4 Gb of memory. The *emm* type for each sample can be identified by searching the gene from the Samples view. The phylogeny tree, on the other hand, can show the relationships between collected samples, highlighted by the MLST to aid the epidemiological investigation. In addition, the heatmap can depict the presence/absence of the virulence genes, such as the superantigens specA/B/C/G/J, for each and every sample in the set.

## Discussion

We have presented AMRViz, a toolkit for analyzing, visualizing, and managing large collections of bacterial genomes. The toolkit is bundled with the current best practice analysis pipeline for bacterial genomics, providing a comprehensive analysis of every single sample and the collection. The analysis results can be visualized via a web-based platform, allowing investigators to interactively examine various aspects of the collection. The complete software package can be easily installed on any common computer system. Both the underlying analysis pipeline and the web application are highly extendable; new analysis tools can be easily incorporated into the pipeline as they become established. We will also include additional visualization tools to support new analyses as required. We anticipate the software to be of general importance in the field of bacterial genomics. The method presented will be a utility for scientists to rapidly investigate bacterial antibiotic resistance.

### Availability of source code and requirements

- Project name: AMRViz.
- Project home page:  https://github.com/amromics/amrviz
- Operating system(s): Platform independent
- Programming language: Python and JavaScript
- License: MIT

### Supplementary Information

**Additional file 1**. Supplementary Figures S1–S7.

## Data Availability

Data used in the manuscript are made available on the project home page https://github.com/amromics/amrviz/tree/main/examples. Scripts to reproduce the analysis are also provided on the GitHub repository.The data results from the case study are available on figshare at 10.6084/m9.figshare.25560645.v1.

## References

[1] Tettelin H, Masignani V, Cieslewicz MJ, Donati C, Medini D, Ward NL, Angiuoli SV, Crabtree J, Jones AL, Durkin AS, DeBoy RT, Davidsen TM, Mora M, Scarselli M, Margarit y Ros I, Peterson JD, Hauser CR, Sundaram JP, Nelson WC, Madupu R, Brinkac LM, Dodson RJ, Rosovitz MJ, Sullivan SA, Daugherty SC, Haft DH, Selengut J, Gwinn M.L, Zhou L, Zafar N, Khouri H, Radune D, Dimitrov G, Watkins K, O’Connor KJB, Smith S, Utterback TR, White O, Rubens CE, Grandi G, Madoff LC, Kasper DL, Telford JL, Wessels MR, Rappuoli R, Fraser CM. Genome analysis of multiple pathogenic isolates of *Streptococcus agalactiae*: implications for the microbial “pan-genome”. Proc Natl Acad Sci. 2005;102:(39)13950–13955. 10.1073/pnas.0506758102. 10.1073/pnas.0506758102 PMC1216834 16172379

[2] Cummins EA, Hall RJ, Connor C, McInerney JO, McNally A (2022). Distinct evolutionary trajectories in the *Escherichia coli* pangenome occur within sequence types. Microb Genom.

[3] McInerney JO, McNally A, O’Connell MJ (2017). Why prokaryotes have pangenomes. Nat Microbiol.

[4] Karlsen ST, Rau MH, Sánchez BJ, Jensen K, Zeidan AA (2023). From genotype to phenotype: computational approaches for inferring microbial traits relevant to the food industry. FEMS Microbiol Rev.

[5] Do VH, Nguyen SH, Le DQ, Nguyen TT, Nguyen CH, Ho TH, Vo NS, Nguyen T, Nguyen HA, Cao MD (2024). Pasa: leveraging population pangenome graph to scaffold prokaryote genome assemblies. Nucleic Acids Res.

[6] De la Fuente J, Diez-Delgado I, Contreras M, Vicente J, Cabezas-Cruz A, Tobes R, Manrique M, Lopez V, Romero B, Bezos J (2015). Comparative genomics of field isolates of *Mycobacterium bovis* and *M. caprae* provides evidence for possible correlates with bacterial viability and virulence. PLoS Negl Trop Dis.

[7] Hendriksen RS, Bortolaia V, Tate H, Tyson GH, Aarestrup FM, McDermott PF (2019). Using genomics to track global antimicrobial resistance. Front Public Health.

[8] Souvorov A, Agarwala R, Lipman DJ (2018). SKESA: strategic k-mer extension for scrupulous assemblies. Genome Biol.

[9] Bankevich A, Nurk S, Antipov D, Gurevich AA, Dvorkin M, Kulikov AS, Lesin VM, Nikolenko SI, Pham S, Prjibelski AD, Pyshkin AV, Sirotkin AV, Vyahhi N, Tesler G, Alekseyev MA, Pevzner PA (2012). SPAdes: a new genome assembly algorithm and its applications to single-cell sequencing. J Comput Biol.

[10] Seemann T (2014). Prokka: rapid prokaryotic genome annotation. Bioinformatics.

[11] Page AJ, Cummins CA, Hunt M, Wong VK, Reuter S, Holden MTG, Fookes M, Falush D, Keane JA, Parkhill J (2015). Roary: rapid large-scale prokaryote pan genome analysis. Bioinformatics.

[12] Jolley KA, Maiden MC (2010). BIGSdb: scalable analysis of bacterial genome variation at the population level. BMC Bioinform.

[13] Carattoli A, Zankari E, Garcia-Fernandez A, Voldby Larsen M, Lund O, Villa L, Moller Aarestrup F, Hasman H (2014). In silico detection and typing of plasmids using PlasmidFinder and plasmid multilocus sequence typing. Antimicrob Agents Chemother.

[14] Feldgarden M, Brover V, Gonzalez-Escalona N, Frye JG, Haendiges J, Haft DH, Hoffmann M, Pettengill JB, Prasad AB, Tillman GE (2021). AMRFinderPlus and the reference gene catalog facilitate examination of the genomic links among antimicrobial resistance, stress response, and virulence. Sci Rep.

[15] Seemann T, G.d.S.A. Github https://github.com/tseemann/nullarbor (2018).

[16] Petit RA, Read TD (2020). Bactopia: a flexible pipeline for complete analysis of bacterial genomes. Msystems.

[17] Schwengers O, Hoek A, Fritzenwanker M, Falgenhauer L, Hain T, Chakraborty T, Goesmann A (2020). ASA3P: an automatic and scalable pipeline for the assembly, annotation and higher-level analysis of closely related bacterial isolates. PLoS Comput Biol.

[18] Le DQ, Nguyen TT, Nguyen CH, Ho TH, Vo NS, Nguyen T, Nguyen HA, Cao MD, Nguyen SH (2024). AMRomics: a scalable workflow to analyze large microbial genome collection. bioRxiv.

[19] Kolmogorov M, Yuan J, Lin Y, Pevzner PA (2019). Assembly of long, error-prone reads using repeat graphs. Nat Biotechnol.

[20] Camacho C, Coulouris G, Avagyan V, Ma N, Papadopoulos J, Bealer K, Madden TL (2009). BLAST+: architecture and applications. BMC Bioinform.

[21] Le DQ, Nguyen TA, Nguyen TT, Nguyen SH, Do VH, Nguyen CH, Phung HT, Ho TH, Nam VS, Nguyen T, Nguyen HA, Cao MD (2023). Efficient inference of large pangenomes with PanTA. Bioarxiv.

[22] Nakamura T, Yamada KD, Tomii K, Katoh K (2018). Parallelization of MAFFT for large-scale multiple sequence alignments. Bioinformatics.

[23] Price MN, Dehal PS, Arkin AP (2010). FastTree 2—approximately maximum-likelihood trees for large alignments. PLoS ONE.

[24] Minh BQ, Schmidt HA, Chernomor O, Schrempf D, Woodhams MD, von Haeseler A, Lanfear R (2020). IQ-TREE 2: new models and efficient methods for phylogenetic inference in the genomic era. Mol Biol Evol.

[25] The HC, Karkey A, Pham Thanh D, Boinett CJ, Cain AK, Ellington M, Baker KS, Dongol S, Thompson C, Harris SR, Jombart T, LeThiPhuong T, Tran Do Hoang N, Ha Thanh T, Shretha S, Joshi S, Basnyat B, Thwaites G, Thomson NR, Rabaa MA, Baker S (2015). A high-resolution genomic analysis of multidrug-resistant hospital outbreaks of *Klebsiella pneumoniae*. EMBO Mol Med.

[26] Larsen MV, Cosentino S, Rasmussen S, Friis C, Hasman H, Marvig RL, Jelsbak L, Sicheritz-Pontén T, Ussery DW, Aarestrup FM, Lund O (2012). Multilocus sequence typing of total-genome-sequenced bacteria. J Clin Microbiol.

[27] Davies MR, Keller N, Brouwer S, Jespersen MG, Cork AJ, Hayes AJ, Pitt ME, De Oliveira DM, Harbison-Price N, Bertolla OM (2023). Detection of *Streptococcus pyogenes* m1uk in Australia and characterization of the mutation driving enhanced expression of superantigen SpeA. Nat Commun.

[28] Butler TA, Story C, Green E, Williamson KM, Newton P, Jenkins F, Varadhan H, van Hal S (2024). Insights gained from sequencing Australian non-invasive and invasive *Streptococcus pyogenes* isolates. Microb Genom.

