# Supplementary material for: AMRViz enables seamless genomics analysis and visualization of antimicrobial resistance
Source: BMC Bioinformatics. 2024 May 16;25:193. doi: 10.1186/s12859-024-05792-9 (PMC11100100; doi:10.1186/s12859-024-05792-9)
Supplement: Supplementary file 1 — Additional file 1. Supplementary Figures S1–S7. [file 12859_2024_5792_MOESM1_ESM.docx]

SUPPLEMENTARY INFORMATION

AMRViz enables seamless genomics analysis and visualization of antimicrobial resistance

| **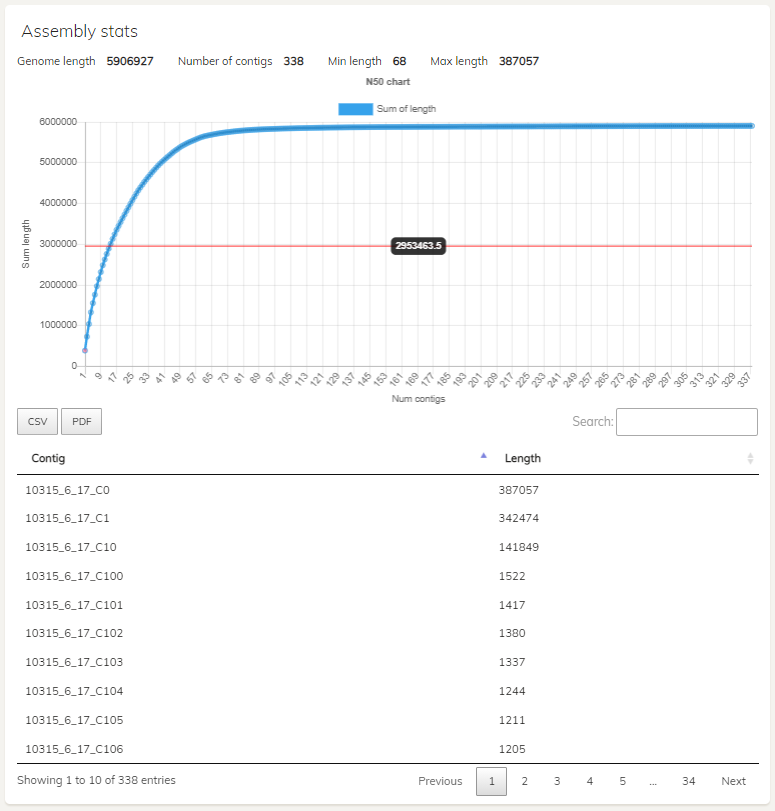 A)** | **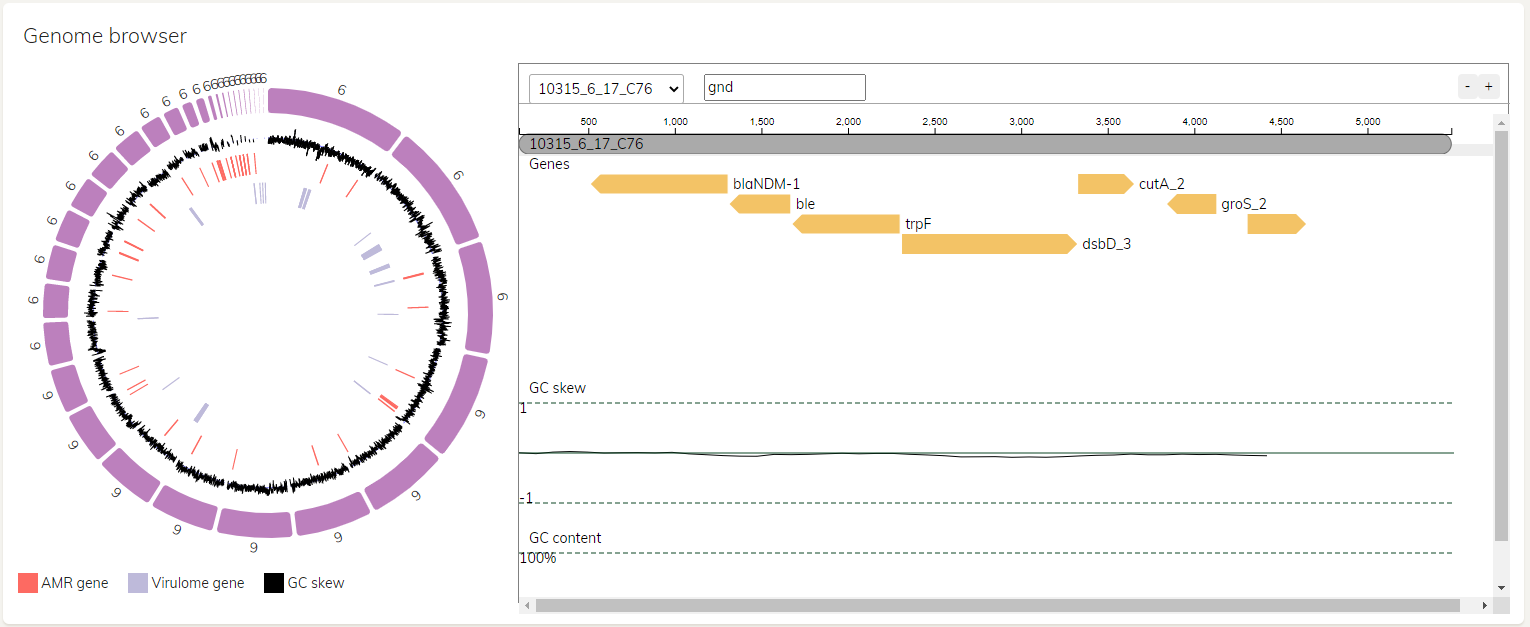**  **B)** | |
| --- | --- | --- |
| **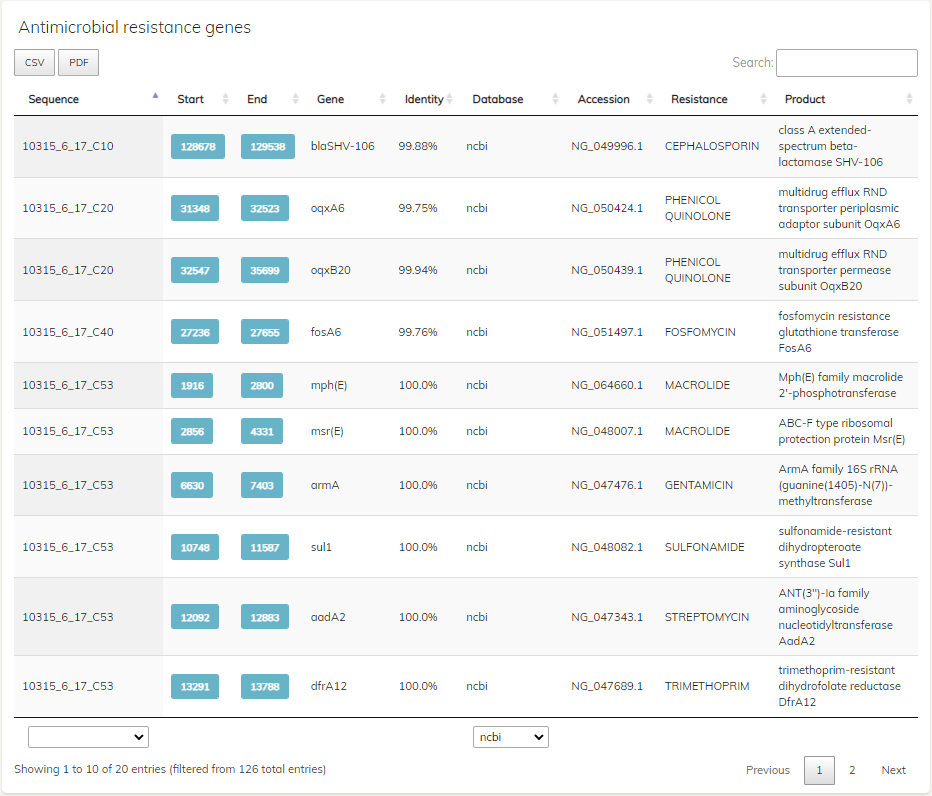**  **C)** | | **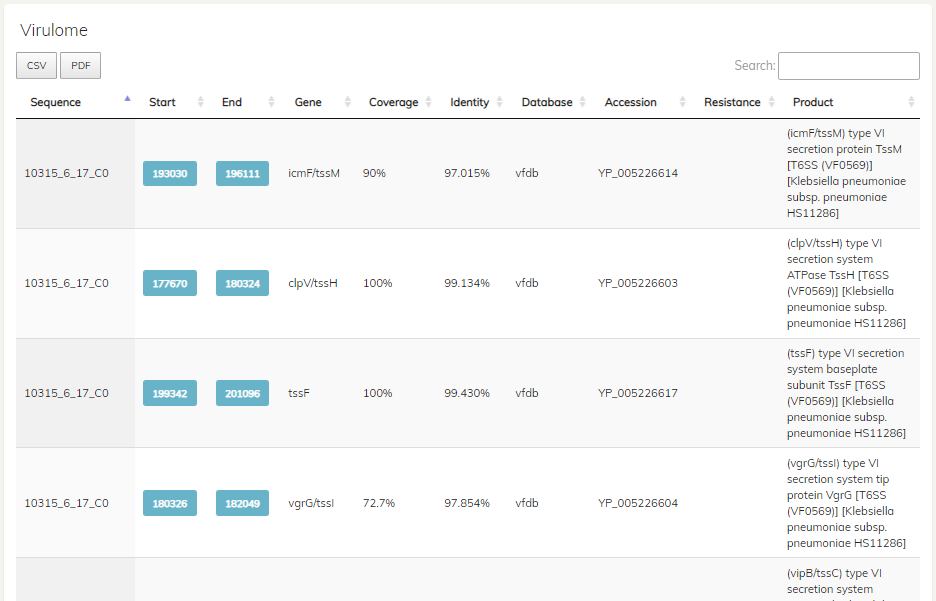**  **D)** |
| **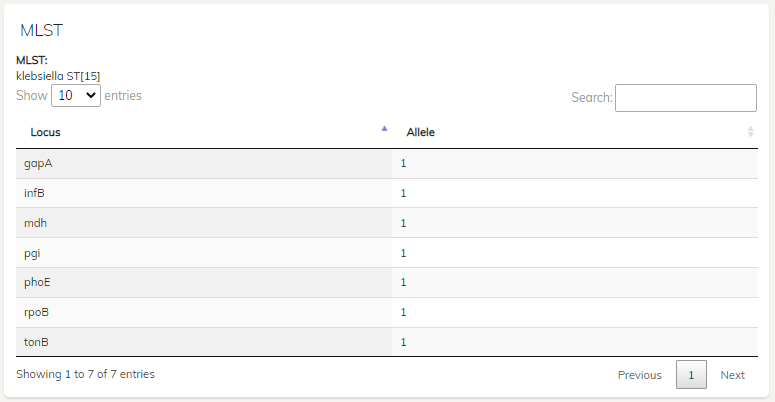**  **E)** | | |

*Figure S1. Visualization of a single sample analysis, using isolate 10315_6#17 (The et al., 2015). A) The statistics of the assembly including N50, cumulative contig lengths, and the lengths of each contig. B) The circos assembly structure and genome browser. The genome browser shows the position of blaNDM-1 gene. C) Listing of resistant genes D) Listing of virulent genes, and E) MLST analysis result.*

**
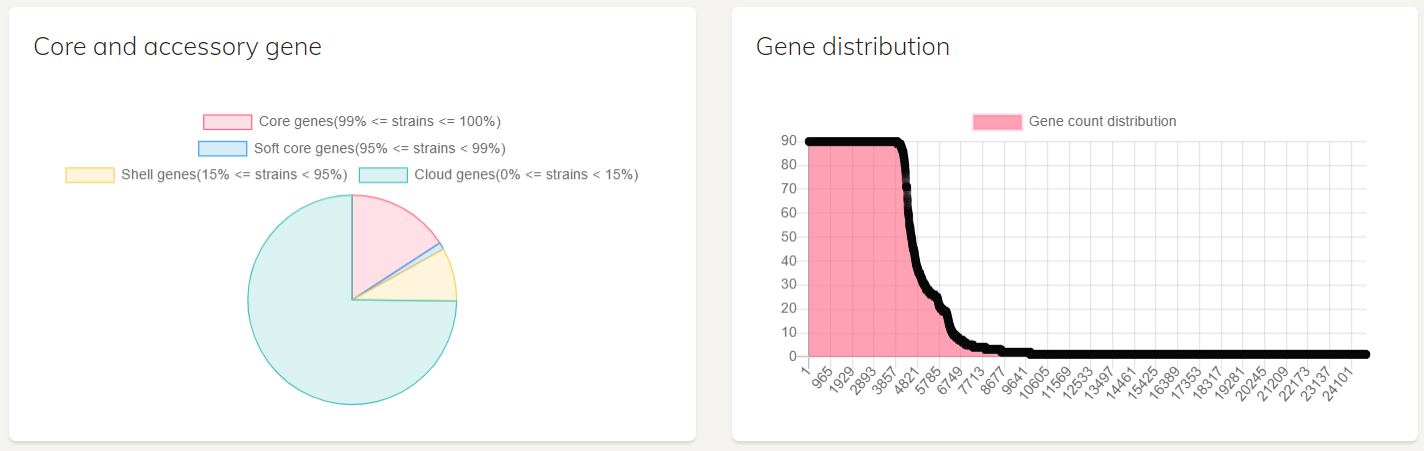
**

*Figure S2. Pan genome summary statistic graphs of 90 klebsiella pneumoniae collection (The et al., 2015)*

**
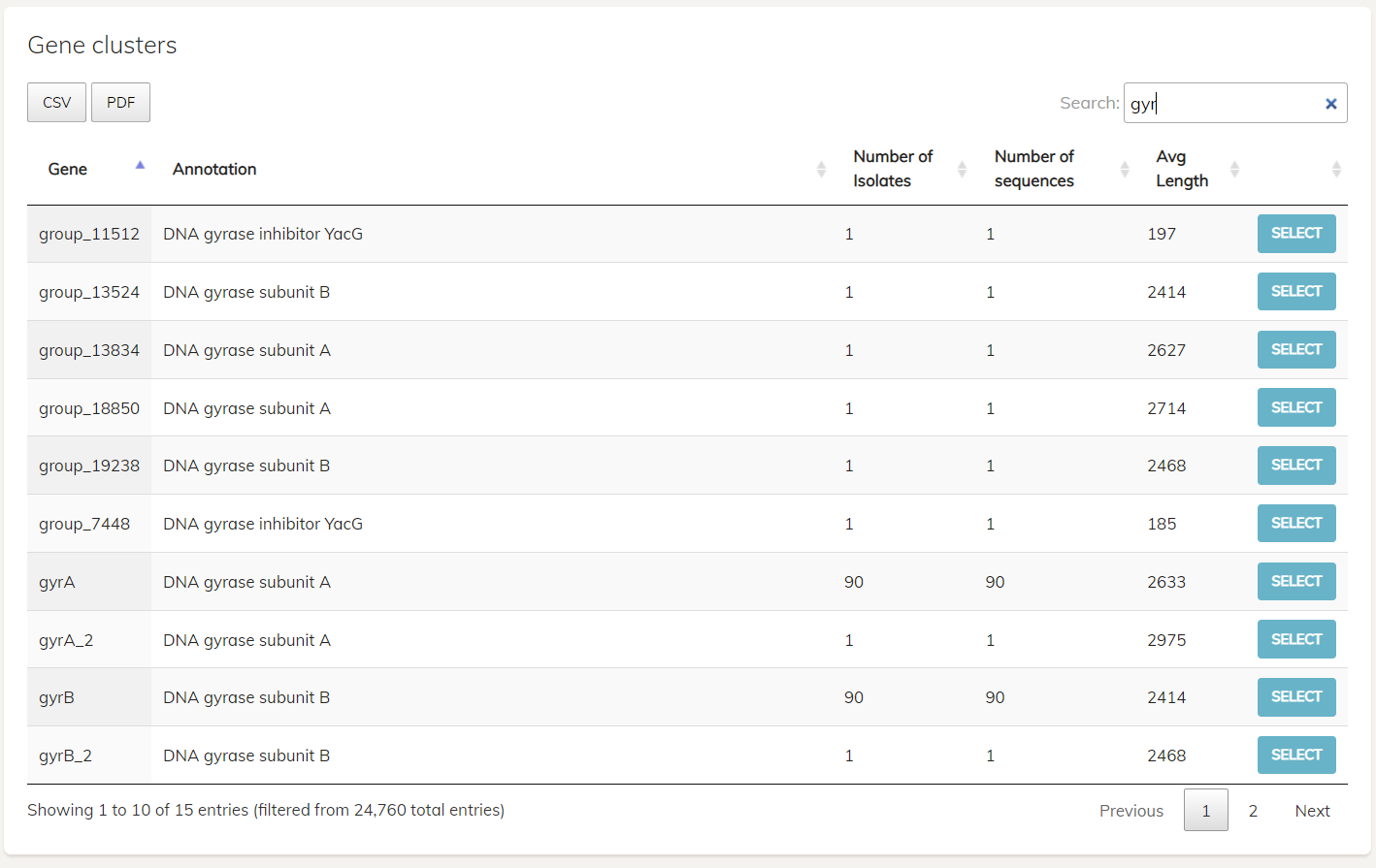
**

*Figure S3. Gene clusters table of 90 klebsiella pneumoniae collection (The et al., 2015)*

**
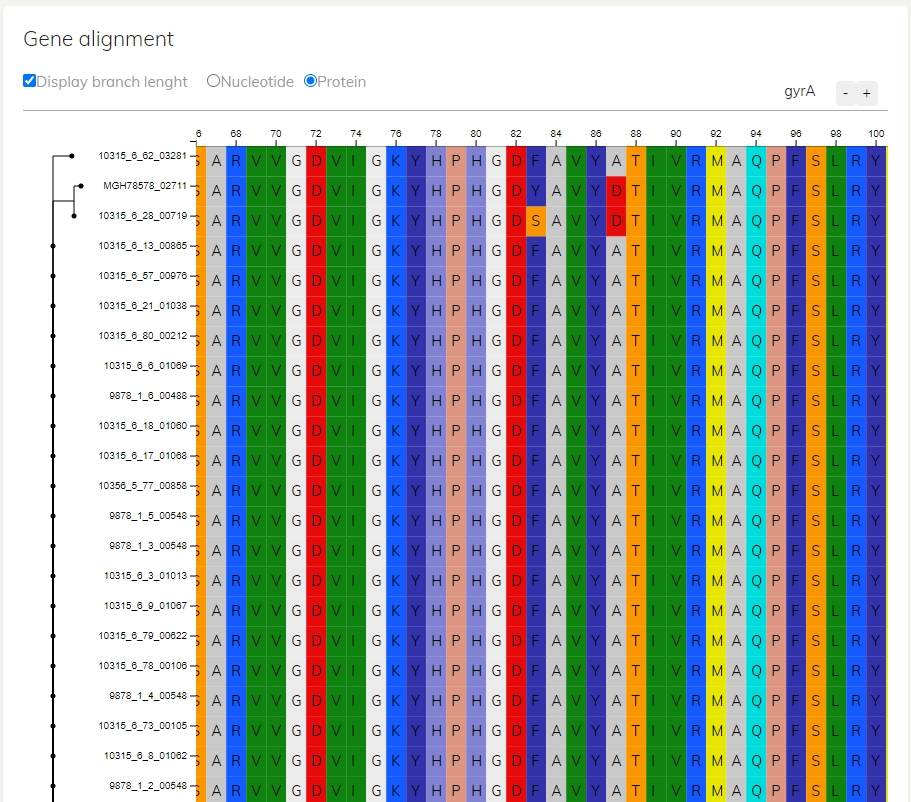
**

*Figure S4 Gene Alignment Viewer shows the multiple alignment of gene gyrA from the isolates in the ST15 group*

**
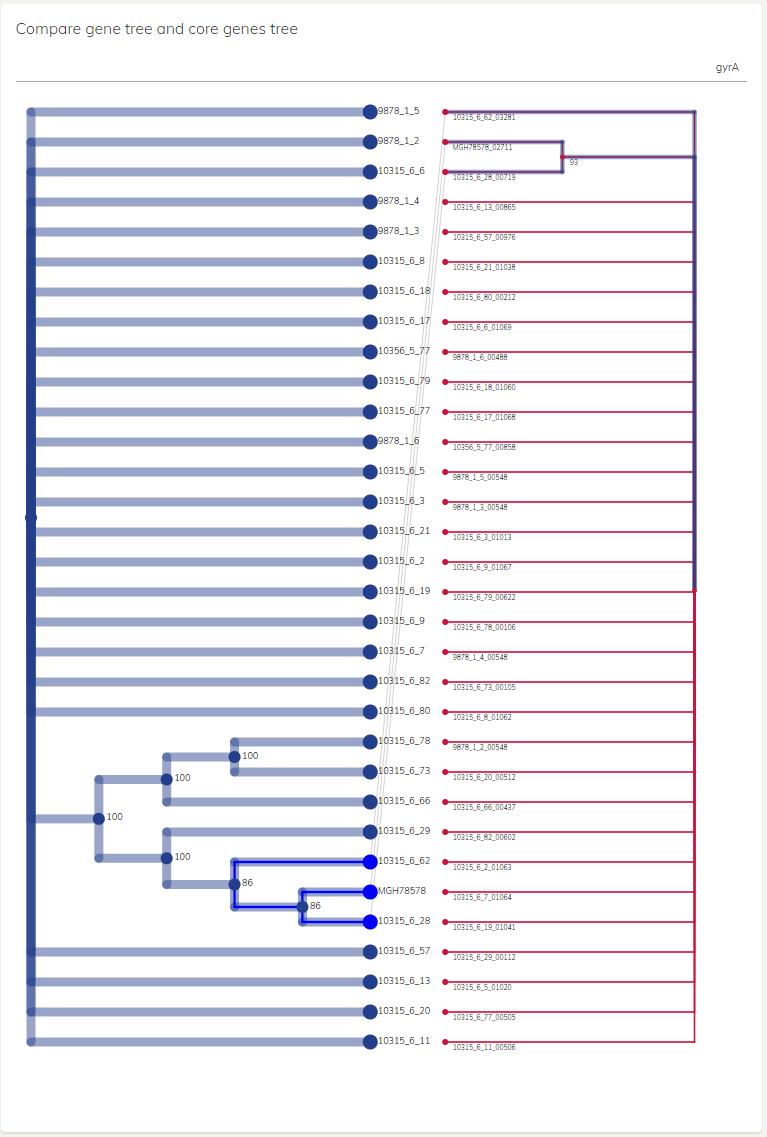
**

*Figure S5. The diagram compares the phylogeny tree of the ST15 isolates and gyrA genes, in which some isolates and their gyrA genes are highlighted.*

**
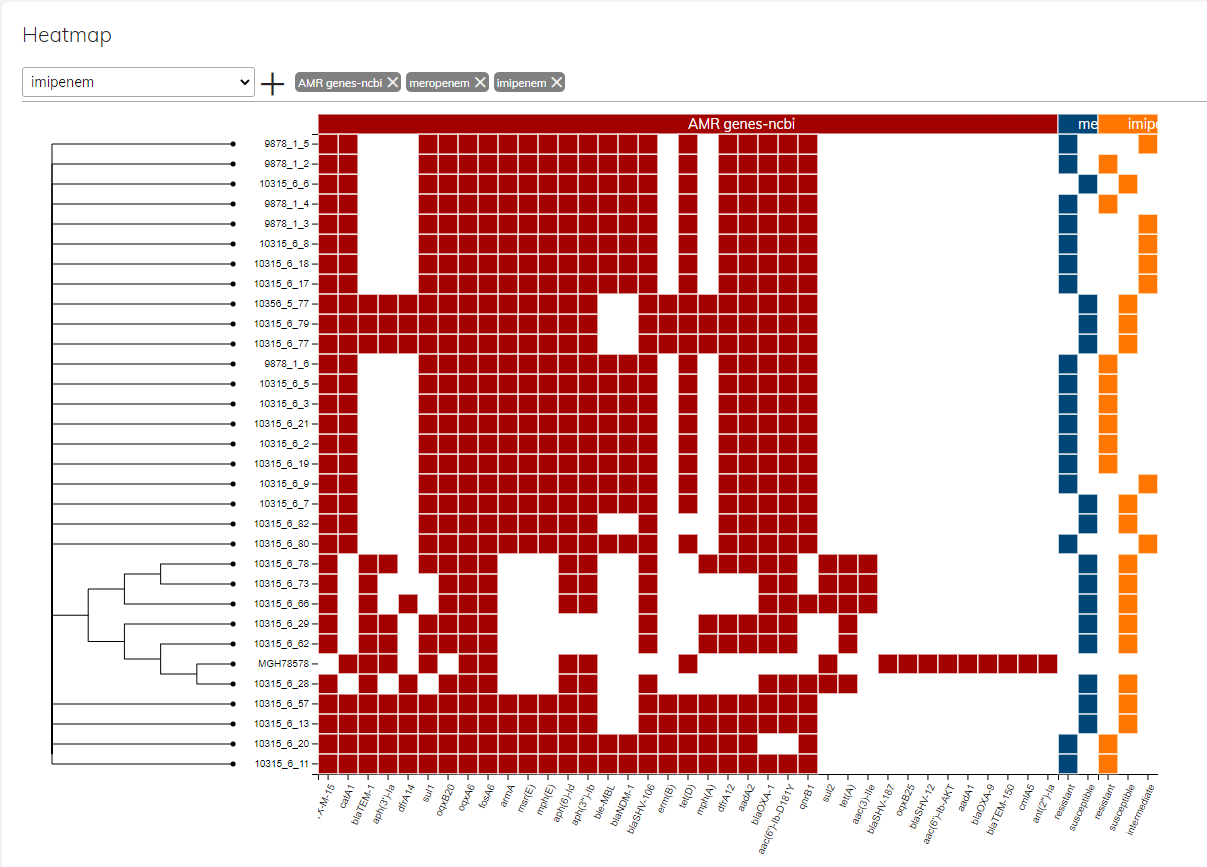
**

*Supplementary Figure 6. The heatmap shows the phylogenetic tree and resistance genes of the ST15 group (The et al., 2015) with the carbapenems antibiotic resistance phenotype including imipenem and meropenem.*

**
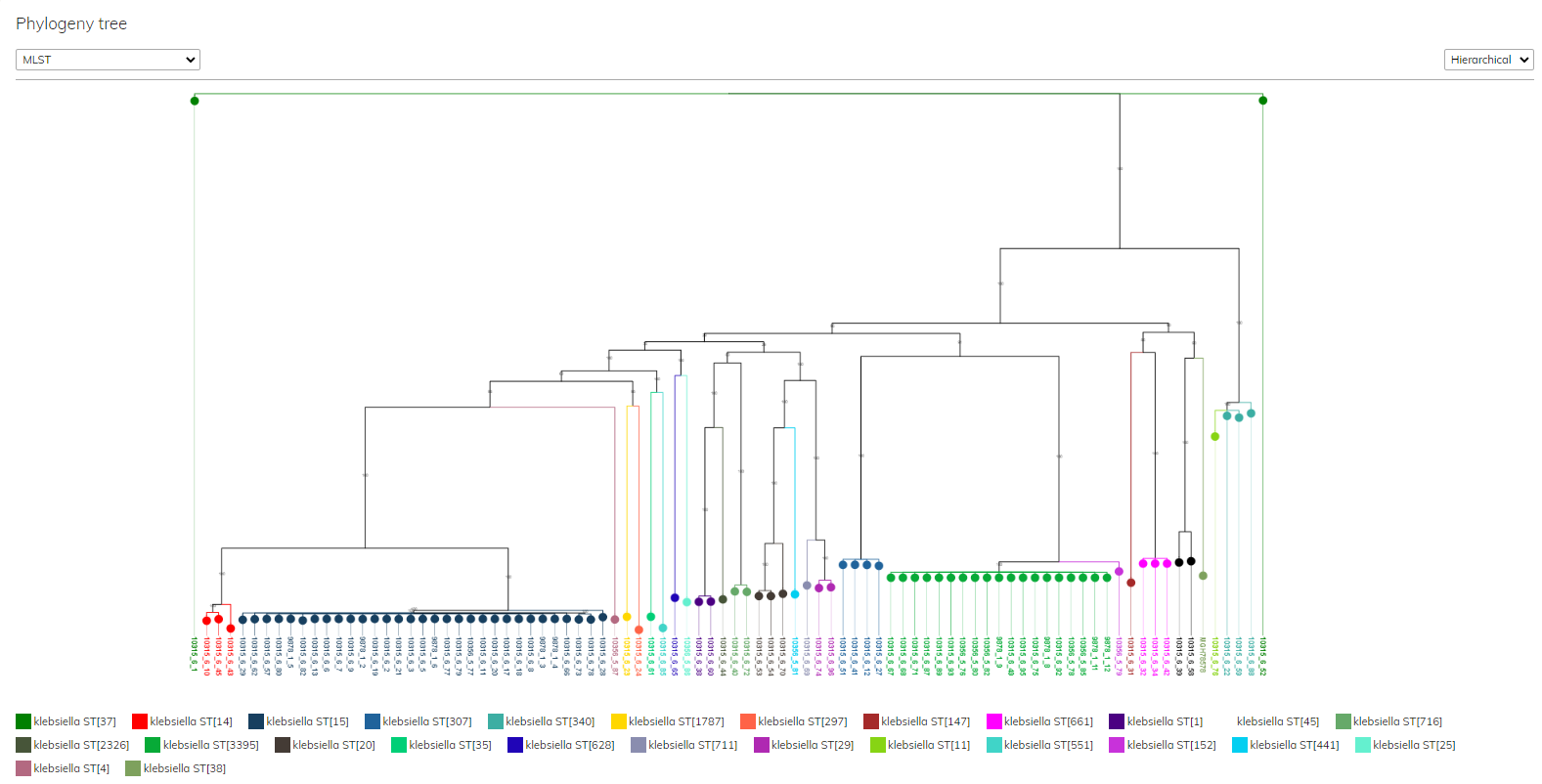
**

*Figure S7. Phylogenetic tree of 90 klebsiella pneumoniae collection (The et al., 2015) with MLST sequence type distinguished by color*
